# Mapping the future: Predictive value of commanded electrogram parameters for the pacing performance in active-fixation leadless pacemakers

**DOI:** 10.1016/j.hroo.2025.07.019

**Published:** 2025-08-05

**Authors:** Takehiro Nomura, Kosuke Onodera, Daiki Kumazawa, Yosuke Mizuno, Kennosuke Yamashita

**Affiliations:** Heart Rhythm Center, Department of Cardiovascular Medicine, Sendai Kosei Hospital, Sendai, Miyagi, Japan

**Keywords:** Bradycardia, Leadless pacemaker, Electrocardiogram, Current of injury, Amplitude, Pacing threshold

## Abstract

**Background:**

In Aveir VR (Abbott Medical) leadless pacemaker implantation, commanded electrograms (cEGMs) are used before and after fixation to evaluate the suitability of the fixation site. However, no clinical studies have examined its predictive value or optimal cutoff values.

**Objective:**

The purpose of this study was to investigate the association between the midterm pacing capture threshold (PCT) and cEGM parameters after Aveir VR implantation procedures.

**Methods:**

We retrospectively analyzed consecutive 74 patients who underwent Aveir VR implantation at Sendai Kosei Hospital with analyzable cEGM data. cEGM parameters, including QRS amplitude, QRS duration, and current of injury (COI), were measured during the premapping and tether modes. The highest PCT recorded within 1 year of implantation was used as the primary outcome. An elevated PCT was defined as >1.5 V/0.4 ms. Correlations were analyzed, and receiver operating characteristic analyses identified optimal cutoff values for the COI and QRS amplitude.

**Results:**

Both the COI and the QRS amplitude showed weak but significant correlations with the PCT. The area under the curve for the COI was 0.715 (premapping) and 0.668 (tether), with cutoff values of >2.0 mV. The COI was associated with a high PCT (premapping mode: odds ratio [OR] 7.5, 95% confidence interval [CI] 1.6–44.6; tether mode: OR 4.8, 95% CI 1.1–21.4). The QRS amplitude had area under the curves of 0.771 in the premapping mode and 0.840 in the tether mode, with cutoff values of >5.0 mV (OR 12.0; 95% CI 1.4–101.7) and >4.0 mV (OR 15.5; 95% CI 2.8–83.9), respectively.

**Conclusion:**

Even before fixation, the COI and QRS amplitude are useful predictors of the midterm PCT after Aveir VR implantation. Combined assessment may enhance fixation strategies.

## Graphical abstract

**Figure:**
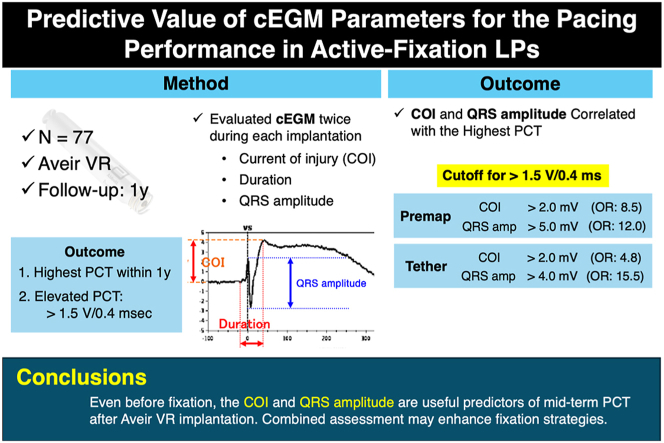

**Key Findings**

▪ Both the current of injury (COI) and the QRS amplitude derived from commanded electrograms were significantly correlated with the midterm pacing capture threshold after Aveir VR (Abbott Medical) leadless pacemaker implantation.
▪ Notably, these parameters were predictive even before fixation (during the premapping phase), highlighting their value in guiding fixation site selection.
▪ Cutoff values of >2.0 mV for the COI and >4.0–5.0 mV for the QRS amplitude may aid in optimizing fixation strategies and reducing the risk of elevated pacing thresholds (>1.5 V/0.4 ms) within 1 year.

## Introduction

Leadless pacemakers (LPs) have emerged as an effective therapeutic option for the management of bradycardia.^1^, ^2^, ^3^, ^4^, ^5^, ^6^, ^7^, ^8^, ^9^, ^10^ The Aveir VR (Abbott Medical) is the only active-fixation LP currently capable of atrial and dual-chamber pacing.^6^^,^^10^ During the implantation procedure, the pacing capture threshold (PCT), impedance, and intracardiac electrograms (EGMs) are assessed prior to the helix fixation and device release. Unlike conventional transvenous pacemakers, however, the real-time intracardiac EGM is not displayed. Instead, a system-defined “commanded EGM” (cEGM) is provided, representing a filtered signal captured within a −100 to +500 ms window around an automatically annotated event. The specific filtering process and annotation algorithms used to generate cEGMs have not been publicly disclosed. In the current Aveir VR implantation procedure, the cEGM is primarily used to evaluate the current of injury (COI), and device manufacturers recommend fixation at sites where the COI amplitude is ≥2.0 mV. However, the validity of this cutoff value, as well as the potential importance of other cEGM parameters, such as amplitude and duration, has not been sufficiently investigated. Retrospective studies have previously shown a significant correlation between the impedance and the PCT in LPs, irrespective of whether an active or passive fixation mechanism was used,^5^^,^^11^^,^^12^ and the clinical utility of the cEGM remains unclear. This study aimed to evaluate the usefulness and optimal cutoff values of the cEGM, thereby providing insights for improving the implantation technique and follow-up strategy for Aveir VR implantation.

## Methods

### Study design

We evaluated the Aveir VR LP system in a retrospective investigator-driven registry. A total of 85 consecutive patients who received an Aveir VR LP between March 2023 and March 2024 at Sendai Kousei Hospital were enrolled, of whom 74 with analyzable cEGM data were included in the final analysis. All cases were followed for a period exceeding 1 year. The study was approved by the Sendai Kosei Hospital institutional review boards.

### Procedural workflow and follow-up

The implantation procedure was performed according to the manufacturer’s recommended workflow. The PCT, impedance, and cEGM were recorded at 3 time points: prior to fixation (premapping), prior to device release (tether mode), and at the end of the procedure. The cEGMs were exported and saved as PDF files from the analyzer. Whenever possible, the LP was fixed according to the manufacturer’s recommendations—at a site where a COI of ≥2 mV, a pacing threshold of ≤1.5 V/0.4 ms, and an increase in the impedance were observed. However, in cases where favorable parameters could not be obtained despite multiple mapping attempts, the final fixation site was determined at the operator’s discretion, even if the criteria were not fully met. Follow-up electrical measurements were conducted within 5 days of implantation, and after 1 month, 3 months, and 1 year. During follow-up, the impedance, sensing, threshold, pacing burden, and predicted battery life were recorded.

### cEGM evaluation

**Figure 1:**
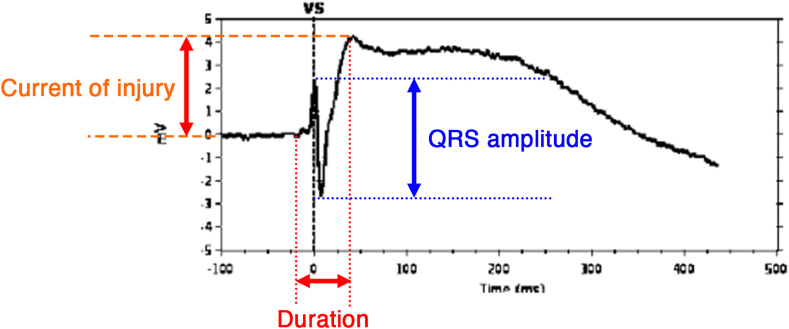
Example of a commanded electrogram (cEGM) measurement. The current of injury amplitude is measured from the baseline to the J point; the duration is from the initial deflection to the J point; the QRS amplitude is the height from the R-wave peak to the S-wave nadir.

cEGMs were saved only as image files, preventing direct measurement on the programmer. Therefore, the PDF version was opened in a PDF reader, and its distance measurement tool was used to assess the amplitudes of the positive and negative waves, EGM duration, and COI. The specific portion of the cEGM used for the measurement is illustrated in Figure 1. Measurements were calibrated using the time (horizontal) and amplitude (vertical) axes shown on the cEGM. The COI was defined as the magnitude of the ST-segment elevation. The *cEGM duration* was defined as the time from the initial deflection to the J point, as the transition between the QRS complex and the ST segment is often indistinct in cEGM recordings and the end of the T wave is frequently unclear.

### Statistical analysis

Continuous variables are expressed as mean ± standard deviation or median with interquartile range, as appropriate, based on the distribution. Categorical variables are presented as count and percentage. Comparisons between the premapping and tether modes were performed using paired *t* tests for continuous variables. The Wilcoxon signed rank test was used to evaluate whether each cEGM parameter differed between the premapping phase and the tether mode. Correlations between continuous variables were assessed using a linear regression analysis, and the coefficient of correlation (*r*) and *P* values are reported. In the present study, the *PCT* was defined as the maximum value recorded during the 1-year observation period. A receiver operating characteristic curve analysis was performed to evaluate the accuracy of the COI and QRS amplitude during the implantation procedure to predict a PCT elevation during the follow-up period. Odds ratios and 95% confidence intervals (CIs) were also calculated. In this cohort, an *elevated PCT* was defined as a value at or above the 90th percentile of the PCT distribution. A 2-tailed *P* value of <.05 was considered statistically significant. All statistical analyses were performed using JMP Pro 17.0 software (SAS Institute Inc.).

### Ethics approval

This study was conducted in accordance with the principles of the Declaration of Helsinki and was approved by the Sendai Kousei Hospital Local Review Board. In accordance with the ethical guidelines for clinical research, an opt-out process was implemented, allowing the patients to decline participation. Details of the study and the opt-out procedure were disclosed on the Web site (https://www.sendai-kousei-hospital.jp) to ensure transparency.

### Outcome

Between March 2023 (corresponding to the first Aveir VR implantation procedure performed at our facility) and April 2024, a total of 85 consecutive patients were enrolled. Of those, 11 patients were excluded: 9 because of the absence of available cEGM data and 2 because of the presence of cEGM recordings obtained during ventricular pacing with the Aveir device. Consequently, 74 patients were included in the final analysis (Table 1). One-year follow-up was successfully completed in 62 patients. Of the 12 patients without completed follow-up, 8 died of noncardiac causes, 2 were transferred to distant facilities, 1 experienced a cardiovascular death because of an acute aortic dissection, and 1 was lost to follow-up. The median (interquartile range) age was 82 years (74–87 years), with 44 male patients (59.4%) and 41 (55.4%) diagnosed with sick sinus syndrome. The median (interquartile range) left ventricular ejection fraction was preserved at 69% (64%–77%). The median (interquartile range) procedure time was 32 minutes (24–40 minutes), with redocking required in 1 patient (1.4%). An arteriovenous fistula was observed in 1 case, but no major complications, including tamponade or dislodgment, occurred. The median (interquartile range) QRS duration and amplitude were 76.5 ms (66.8–88.0 ms) and 5.5 mV (3.5–7.6 mV) during the premapping phase and 85.0 ms (74.8–96.3 ms) and 7.0 mV (4.0–9.1 mV) during the tether mode, respectively (Table 2). Those values tended to be higher during the tether mode, reaching statistical significance (*P* < .001 and *P* = .0014, respectively). Furthermore, the COI amplitude was 3.0 mV (2.0–4.5 mV) in the premapping phase and 3.5 mV (2.0–6.0 mV) in the tether mode; however, that difference was not statistically significant (*P* = .18). The median (interquartile range) impedance was significantly higher in the tether mode at 630 Ω (478–803 Ω), compared with 410 Ω (378–473 Ω) in the premapping phase (*P* < .001).

**Table 1:** Baseline characteristics and procedural variables (N = 74)

Characteristic	Value
Clinical characteristics	
Age (y)	82 (74–87)
Men	44 (59.4)
Weight (kg)	54.4 (48.0–67.1)
Height (cm)	158.7 (150.6–166.6)
Body surface area (m^2^)	1.13 (1.0–1.34)
Hypertension	47 (63.5)
Diabetes	24 (32.4)
Atrial fibrillation	
Paroxysmal	24 (32.4)
Persistent	20 (27.0)
Ischemic heart disease	6 (8.1)
Myocardial infarction	4 (5.4)
Congestive heart failure	33 (44.6)
Indication	
Sinus node dysfunction	41 (55.4)
Atrioventricular block	33 (44.6)
Oral medicine	
Antiarrhythmic drug	
Class I	7 (9.6)
Class III	1 (1.4)
Class IV	3 (4.1)
β-Blocker	16 (21.6)
Angiotensin receptor–neprilysin inhibitor	8 (10.8)
Renin-angiotensin system inhibitor	30 (40.5)
Mineralocorticoid receptor antagonist	10 (13.5)
Sodium-glucose cotransporter 2 inhibitor	12 (16.2)
Diuretics	25 (33.8)
Calcium blocker	37 (50.0)
Echocardiographic data	
Ejection fraction (%)	69 (64–77)
LVEDD (mm)	45 (41–49)
LVESD (mm)	26 (23–30)
IVST (mm)	12 (10–14)
LVPWT (mm)	10 (9–12)
Right ventricular short-axis diameter (mm)	27 (25–30)
Right atrial short-axis diameter (mm)	35 (32–41)
Right atrial long-axis diameter (mm)	55 (49–62)
TRPG (mm Hg)	27 (27–32)
Procedural variables	
Procedure time (min)	32 (24–40)
Fluoroscopy time (min)	13.3 (7.9–19.5)
Redocking	1 (1.4)
Complications	
Tamponade	0 (0)
Pericardiac effusion	0 (0)
Arteriovenous shunt	1 (1.4)
Dislodgment	0 (0)

Values are presented as median (interquartile range) or n (%).

IVST = interventricular septum thickness; LVEDD = left ventricular end-diastolic diameter; LVESD = left ventricular end-systolic diameter; LVPWT = left ventricular posterior wall thickness; TRPG = tricuspid regurgitation pressure gradient.

**Table 2:** Commanded electrograms and electrical measurements

Characteristics	Premapping mode (n = 74)	Tether mode (n = 74)	*P*
On external pacing	10 (13.5)	10 (13.5)	N/A
Commanded EGM			
QRS duration (ms)	76.5 (66.8–88.0)	85.0 (74.8–96.3)	<.001
QRS amplitude (mV)	5.5 (3.5–7.6)	7.0 (4.0–9.1)	.0014
Current of injury (mV)	3.0 (2.0–4.5)	3.5 (2.0–6.0)	.18
Current of injury ≤ 2.0 mV	26 (35.1)	25 (33.8)	.13
Electrical measurement			
Impedance (Ω)	410 (378–473)	630 (478–803)	<.001
Amplitude (mV)	6.5 (4.5–8)	6.5 (4.5–10.3)	.086
PCT at a 0.4ms-pulse width	1.0 (0.75–1.5)	1.0 (0.5–1.5)	.41

Values are presented as median (interquartile range) or n (%).

EGM = electrogram; N/A = not applicable; PCT = pacing capture threshold.

**Figure 2:**
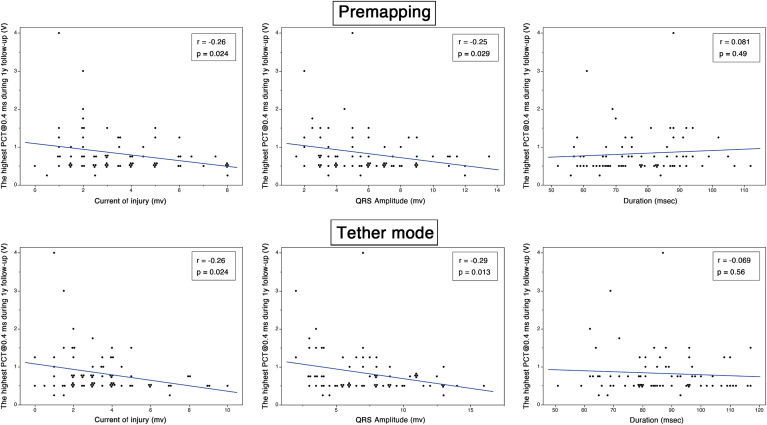
Relationships between the highest pacing capture threshold (PCT) during 1-year follow-up and electrogram parameters in the premapping and tether modes. Scatter plots demonstrate the correlations between the PCT at a 0.4-ms pulse width and (*left*) the current of injury (COI), (*middle*) QRS amplitude, and (*right*) QRS duration, assessed in both the premapping (*top row*) and tether (*bottom row*) modes. Weak but statistically significant inverse correlations were observed between the PCT and the COI (both modes: *R*^2^ = 0.069, *P* = .024) and between the PCT and the QRS amplitude (premapping mode: *R*^2^ = 0.064, *P* = .029; tether mode: *R*^2^ = 0.082, *P* = .013). No significant correlations were found between the PCT and the QRS duration (premapping mode: *R*^2^ = 0.0066, *P* = .49; tether mode: *R*^2^ = 0.0048, *P* = .56).

The COI and PCT exhibited a weak but significant correlation in both the premapping and tether modes (*r* = −0.26; *P* = .024) (Figure 2). A similarly weak but significant correlation was found between the PCT and the QRS amplitude (premapping mode: *r* = −0.25, *P* = .029; tether mode: *r* = −0.29, *P* = .013). In contrast, no significant correlation was observed between the PCT and the QRS duration (premapping mode: *r* = 0.081, *P* = .49; tether mode: *r* = −0.069, *P* = .56). Of the 11 cases in which the COI amplitude was exactly 2.0 mV during the premapping phase, 5 exhibited an elevated PCT exceeding 1.5 V/0.4 ms. Similarly, in the tether mode, 3 of the 11 cases with a COI amplitude of 2.0 mV demonstrated elevated thresholds (Figure 2).

**Figure 3:**
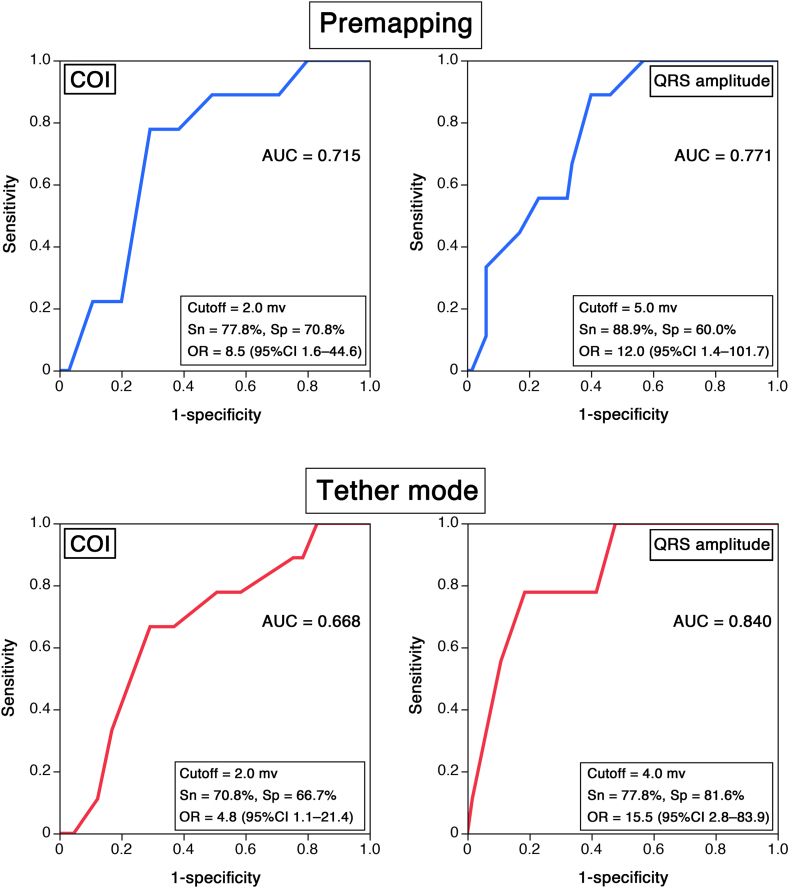
Receiver operating characteristic curve analyses were performed to evaluate the predictive accuracy of the current of injury (COI) and QRS amplitude during the premapping phase for an elevated pacing capture threshold (≥1.5 V/0.4 ms). The areas under the curves were 0.715 for the COI and 0.771 for the QRS amplitude. The optimal cutoff values determined using the Youden index were 2.0 mV (COI: sensitivity [Sn] 77.8%; specificity [Sp] 70.8%; odds ratio [OR] 8.6; 95% confidence interval [CI] 1.6–44.6) and 5.0 mV (QRS: Sn 88.9%; Sp 60.0%; OR 12.0; 95% CI 1.4–101.7). During the tether mode, the area under the curves (AUCs) were 0.668 for the COI and 0.840 for the QRS amplitude. The optimal cutoff values were 2.0 mV (COI: Sn 70.8%; Sp 66.7%; OR 4.8; 95% CI 1.1–21.4) and 4.0 mV (QRS: Sn 77.9%; Sp 81.6%; OR 15.5; 95% CI 2.8–83.9).

Receiver operating characteristic curve analyses (Figure 3) were performed to evaluate the predictive accuracy of the COI and QRS amplitude during the premapping phase for an elevated PCT (≥1.5 V/0.4 ms). The area under the curves were 0.715 for the COI and 0.771 for the QRS amplitude. The optimal cutoff values determined using the Youden index were 2.0 mV (COI: sensitivity 77.8%; specificity 70.8%; odds ratio 8.6; 95% CI 1.6–44.6) and 5.0 mV (QRS: sensitivity 88.9%; specificity 60.0%; odds ratio 12.0; 95% CI 1.4–101.7). During the tether mode, the area under the curves were 0.668 for the COI and 0.840 for the QRS amplitude. The optimal cutoff values were 2.0 mV (COI: sensitivity 70.8%; specificity 66.7%; odds ratio 4.8; 95% CI 1.1–21.4) and 4.0 mV (QRS: sensitivity 77.9%; specificity 81.6%; odds ratio 15.5; 95% CI 2.8–83.9).

## Discussion

Several noteworthy findings emerged from the present analysis. First, the COI, particularly during the premapping phase, was associated with an increase in the PCT of the Aveir VR up to 1-year postimplantation. The COI has been previously reported as a useful parameter for predicting the fixation and performance of active fixation transvenous leads.^13^, ^14^, ^15^, ^16^, ^17^ Studies analyzing the COI in EGMs obtained via actively fixed leads have shown that a higher COI is associated with improvements in pacing thresholds over time.^13^ Interestingly, the COI observed in active-fixation leads—both in the right atrium and in the right ventricle (RV)—has also been shown to be a useful predictor of midterm favorable pacing thresholds, consistent with the present findings in the Aveir VR system.^14^^,^^18^ In contrast, an observational study of 1011 patients reported that an increase in PCT was not associated with device-related characteristics at implantation, including RV sensing, impedance, and initial PCT.^19^ More recently, several studies have highlighted the utility of monitoring the COI during a deep septal lead placement for left bundle branch area pacing.^20^, ^21^, ^22^, ^23^, ^24^, ^25^ However, the Aveir VR LP differs from transvenous systems in several respects: the helix is not an electrode, the distance between the cathode and the anode is longer, and the electrode surface area is larger. In myocardial ischemia, the COI is primarily generated because of differences in the resting membrane potentials between injured and noninjured tissue, with the injured region typically exhibiting partial depolarization.^26^ Additionally, ischemic myocardium often undergoes earlier repolarization, which can give rise to systolic injury currents.^27^^,^^28^ However, the observation that the COI can be detected even prior to the helix fixation with the Aveir VR device suggests that true tissue damage may not be necessary; rather, mechanical compression alone may be sufficient to alter the resting membrane potential or action potential characteristics. Furthermore, the prefixation COI may reflect intrinsic myocardial properties, such as tissue viability or mechanical responsiveness, and could potentially serve as an indirect marker of myocardial health. While our study did not directly evaluate tissue characteristics, this hypothesis may partly explain the predictive value of the COI prior to deployment. With the Aveir system, the region injured by the helix is not the myocardium directly in contact with the electrode, but rather the surrounding tissue. Additionally, the internal filtering settings are not disclosed, which may alter the clinical implications of the COI. Nevertheless, the COI derived from the cEGM proved to be a useful predictor of a future PCT, even in the context of the Aveir VR system.

Unlike impedance, which typically increases after myocardial fixation, the COI did not exceed the prefixation levels postfixation in our analyses. With LPs, an increase in the postfixation impedance has been shown to be correlated with a lower PCT, likely reflecting healthy myocardium, a large electrode-tissue interface, and a stable fixation. In contrast, the COI does not appear to rise proportionally with increased contact force. Few studies have examined the correlation between the contact force and the COI at the electrode-myocardium interface. One study using unipolar EGMs from ablation catheters showed only a limited correlation between the contact force and the COI amplitude.^29^ This may align with the observation that the COI increased less than the impedance upon the myocardial fixation of the Aveir device. However, as the cEGM is bipolar and likely filtered differently, it is unclear whether this reflects the same phenomenon. Although the postfixation impedance is already recognized as a useful marker for predicting the pacing performance, the present findings suggested that a COI of >2.0 mV during the premapping phase may serve as a valuable indicator even before fixation. Given that redocking may increase the risk of complications, the ability to anticipate fixation success predeployment is clinically meaningful. Although a COI threshold of ≥2.0 mV is currently recommended,^30^ the present cohort analysis indicated that a COI amplitude of just 2.0 mV may not be adequate. Rather, achieving a distinctly suprathreshold COI—clearly exceeding 2.0 mV—was associated with more favorable pacing thresholds.

In addition to the COI, the QRS amplitude was associated with the PCT. While some studies on transvenous RV pacing have shown a correlation between the PCT and the R-wave amplitude, others have found no such relationship.^19^^,^^31^ Although the cEGM amplitude does not necessarily match the surface R-wave amplitude, a larger QRS component may be favorable. However, while the QRS amplitude can be measured with a relatively high accuracy, in some cases, determining the J point can be challenging, so the COI amplitude may be measured inaccurately. This potential inaccuracy could affect the utility of the COI, and therefore, it cannot be conclusively stated that the QRS amplitude is a superior indicator. That said, the QRS amplitude may serve as a more straightforward parameter than the COI. Unlike the COI, the QRS amplitude significantly increased in the tether mode; however, the cutoff value was lower in the tether mode (4.0 mV) than in the premapping phase (5.0 mV). This discrepancy likely reflected the nature of the Youden index, and whether those cutoff values are appropriate warrants further investigation.

Finally, both the cEGM and the COI are likely to be important in Aveir AR implantation procedures as well. Given the greater importance of the cEGM in the implantation of the Aveir AR system, the demonstrated utility of the cEGM in the Aveir VR system is a promising and supportive finding. However, since the helix serves as the cathode in the AR model and the cEGM waveforms tend to be more uniform, a dedicated analysis specific to the AR system is warranted.

### Limitations

This study was a single-center retrospective analysis. cEGM data were available only as image files and could not be imported into an electrophysiology recording system. As a result, all measurements were performed manually using a PDF viewer, which may have yielded different results compared to studies using programmer-based or recording system–based measurements. In addition, the interobserver variability may have influenced the measurements. To minimize such bias, multiple experienced cardiac electrophysiologists independently performed the measurements and reached a consensus through discussion. It is also possible that the optimal cutoff value for the COI may have varied depending on the desired target PCT. However, the definition of a PCT elevation in this study was based on the percentile of the cohort data and previous studies,^11^^,^^32^^,^^33^ rather than being arbitrarily determined.

## Conclusion

The cEGM parameters, particularly the COI and QRS amplitude, are useful predictors of the midterm PCT after Aveir VR implantation, even before fixation. A COI amplitude strictly >2.0 mV during the premapping phase was associated with more favorable pacing outcomes, and the QRS amplitude demonstrated an even higher predictive accuracy, especially during the tether mode. Those findings suggested that not only the COI but also the QRS component of the cEGM should be considered when selecting optimal fixation sites. Further studies are warranted to validate the proposed cutoff values.
